# Illegal urban wild meat supply chain characterization: A case study on zoonosis in Lusaka, Zambia

**DOI:** 10.1371/journal.pone.0342396

**Published:** 2026-02-11

**Authors:** Batsirai Alexander Mukanganwa, Mercy Mukuma, John Shindano, Himoonga Bernard Moonga

**Affiliations:** Department of Food Science and Nutrition, University of Zambia, Lusaka, Zambia; Public Library of Science, UNITED KINGDOM OF GREAT BRITAIN AND NORTHERN IRELAND

## Abstract

**Introduction:**

The illegal wild meat trade poses significant threats to biodiversity and public health. In Zambia, data regarding trade remain limited. This study aimed to analyze its supply chain, perform a broad risk assessment of multiple zoonotic pathogens, and develop a sustainable wild meat trade framework.

**Methods:**

A grounded theory–based qualitative study was conducted in Lusaka district between October 2023 and February 2024. Thirty-eight in-depth interviews were carried out with key informants from the government, conservation organizations, academia, butchers, and research sectors. The participants were selected using purposive sampling followed by snowballing. The inclusion criterion was individuals with professional or operational knowledge of the wild meat trade; the exclusion criterion included a lack of relevant expertise or refusal to provide informed consent. The interviews were conducted in English or Nyanja; Nyanja interviews were then translated to English. Interviews were transcribed using Whisper AI, and thematically analyzed using grounded theory by means of NVivo 12.

**Results:**

The illicit supply chain includes poachers in protected areas, urban-based financiers, middlemen, and urban consumers. The species most commonly traded include buffalo, kudu, impala, warthog, and hippopotamus. The key zoonotic risk factors identified were unhygienic field processing, transport without refrigeration over distances >300 km, species misrepresentation (e.g., anthrax-infected hippopotamus sold as buffalo), and meat consumption from dead or snared animals. Poor enforcement and high demand in Lusaka sustain the trade. A sustainable wild meat trade framework was developed, focusing on community engagement, alternative livelihoods, game farming, and public health education.

**Conclusion:**

Zambia’s illegal wild meat trade poses serious zoonotic risks due to unhygienic practices, poor oversight, and species misrepresentation. Reducing these risks requires multisectoral collaboration, stronger enforcement, behavior change strategies, and investment in sustainable livelihood alternatives for communities near protected areas.

## 1. Introduction

The demand for wildlife and derived products has increased substantially, exacerbating the illegal wildlife trade [1]. The illegal wildlife trade involves the unauthorized commerce of wild animals and plants as well as their derivatives (wild meat, ivory, rhino horns, and fur) [2]. Globally, it has a financial value estimated to be worth up to US$23 billion annually [2,3].

There is increasing evidence of illegal hunting throughout Africa for the wild meat trade [4,5]. This trade encompasses consumption in rural communities and urban communities [6]. Its value is likely to exceed USD 1 billion per year and possibly several times that amount, with an estimated volume of 1–5 million metric tonnes (mt) [7]. McNamara et al. [8] estimated that informal domestic trade in wild meat within sub-Saharan African countries is worth hundreds of millions of dollars. The wild meat trade has long been recognized as a serious problem in the forest biomes of Africa [9,10]. It is considered a great and immediate threat to biodiversity conservation in Africa [9,11–13].

In Zambia, poaching for wild meat is among the most damaging human activities to wild animal populations [14]. It is one of the greatest threats to the game ranching industry [15]. Illegal wild meat in Zambia comes from national parks (NPs), game management areas (GMAs), and game ranches/farms [15,16]. According to a 2021 Wildlife Crime Prevention report, the DNPW seized 50,244 kilograms of wild meat between 2016 and 2021 [17]. The weight is likely greater considering that much of the illegally traded wild meat is not intercepted.

The illegal wild meat trade has received considerable global attention as a result of the persistent outbreak of zoonotic diseases connected to wildlife and wildlife product commerce [18,19]. This is because, in the illegal wild meat trade, there is unhygienic handling [20], poor transport conditions, avoidance of quarantine controls, and black market trade outside regulated markets and retail outlets where health inspections are not performed [21,22], bypassing health inspections [23], contact with novel or emerging pathogens [24], and mixing of species in transport and markets [25]. Making illegal wild meat trade a gateway for zoonotic agents [1,2].

The COVID-19 pandemic heightened awareness of the potential role of the trade of wild animals and the consumption of wild meat [7]. Zoonosis is not only a health problem but also an economic problem, with estimated global economic costs of COVID-19, ranging from US$77 billion to US$2.7 trillion, and an estimated years of life lost (YLLs) as high as 4 072 325 in 30 high-incidence countries in the first year of the pandemic [26]. Another example is the Severe Acute Respiratory Syndrome (SARS) outbreak, which was linked to civet trade in 2003, which cost China’s economy $25.3 billion and reduced the gross domestic product (GDP) across East Asia by 2% [20,27]. Indicating how costly the wildlife product trade can be.

In the last and present decade, there have been outbreaks linked to wild meat in Africa [28,29]. Ebola virus outbreaks in Guinea, Liberia, and Sierra Leone have had estimated economic impacts ranging from $30 billion to $50 billion [30]. In 2024, the World Health Organization (WHO) declared mpox a public health emergency of international concern due to a surge in cases in the Democratic Republic of the Congo (DRC) and other parts of Africa [31]. It was estimated that $245 million was needed to combat the growing mpox outbreak in Africa [29].

Cases of people falling ill after consuming wild meat infected with anthrax have been reported in Zambia [32,33]. An outbreak of anthrax [32], which is caused by *Bacillus anthracis*. Anthrax is a priority zoonotic disease in Zambia [34]. The outbreak killed 85 *Hippopotamuses amphibious* in the Luangwa Valley in 2011 [35]. A total of 521 human cases and four deaths were recorded, and these cases were linked to illegal wild meat [35]. Following the same outbreak, Lehman et al. [32] reported how supply chain activities from sourcing to consumption were linked to the anthrax infection risk ratio. The study reported skinning hippopotamus (odds ratio 13.3, 4.4–41.5), cutting the meat (odds ratio 8.9, 95% 2.5–47.5), carrying the meat (odds ratio 5.3, 95% 2.0–15.4), preparing (odds ratio 3.3, 95% CI 1.1–13.7), and eating (8.8, 95% CI 1.3–369.3). This study demonstrates a data-based linkage between wild meat supply chain activities and the risk of zoonotic diseases.

There is also the risk of introducing zoonotic disease into animal populations due to illegal wild meat hunting [2,22], which can be very detrimental to communities that rely on livestock. A study by Banda et al. [36] modelled the societal burden of anthrax in cattle in the Western Province of Zambia. The model demonstrated that a cow, bull and ox lost approximately 34%, 39% and 37% of the productivity years of its life span, respectively, due to anthrax. The same study reported that anthrax caused a total loss of 459,280.90 productivity-adjusted life years in the three districts in the western province of Zambia. In another study, Mwacalimba et al. [36] performed a model focusing on the cost‒benefit analysis of tuberculosis (TB) control, which is caused by the wildlife‒livestock interface in areas of southern Zambia. Tuberculosis is another example of priority zoonotic disease in Zambia [33] and is caused by *Mycobacterium bovis*. In this model, avoiding an average of 1300 cases of zoonotic bovine tuberculosis in humans would translate to $152,000 savings in the fourth year and $177,000 by the tenth year, assuming an effective public awareness campaign. The same study estimated that, potentially, 50 calves are foregone annually as a result of bovine tuberculosis in cattle herds, and over $43,000 worth of milk are also lost annually [36]. These diseases are also transboundary bovine tuberculosis [37] and anthrax [23], which can affect neighboring countries in the region. These findings indicate that zoonotic spillover can have an impact on Zambia as a result of the illegal wild meat trade.

Considering that only a proportion of illegal trade is intercepted and acknowledging that the status of illegally traded wildlife and wildlife products may pose a greater risk to public health [38] and economic losses. It is prudent to focus not only on legal trade but also on illegal wildlife trade when trying to lessen the risk of the introduction of zoonotic pathogens [39]. This is even more important since illegally sourced wild meat, in some cases, ends up being sold as legally sourced meat [21,40]. Kock and Caceres-Escobar [3] highlighted that a dearth of data warrants improved surveillance of zoonotic cases attributed to wildlife and the wildlife trade, both legal and illegal. Despite the increasing recognition of these risks, little is known about the structure and practices of illicit wild meat supply chains in Zambia. Hence, the objectives of this study were supply chain analysis of illegal wild meat, broad risk assessment for multiple zoonotic pathogens, and the development of a framework that can be used for sustainable wild meat trade management. Different terminology is used to describe meat that comes from wildlife or game animals. These terms are game meat [41], wild meat [20], wild game meat [42], and bush meat [43]. In Zambia, the most common term is game meat; in some of the published literature, wild meat and bushmeat are used, especially if the meat is sourced informally [44,45]. Hence, to capture both the Zambian and the international research community, in this study, the terms game meat and wild meat are used synonymously.

## 2. Methodology

### 2.1. Study area

The study was conducted in the Lusaka district, which is one of the six districts in the Lusaka province, Zambia. It is also the capital of Zambia, where illegal wild meat is consumed the most, with an estimated annual consumption of 1140 tons [16]. It is the largest city in Zambia. It has an estimated population of approximately 3 million [46]. It is located at −15.41° latitude and 28.29° longitude and is situated at an elevation of 1277 m above sea level.

### 2.2. Approach and design

Primary data were collected from October 2023 to February 2024. A grounded theory–based qualitative study [47,48] composed of in-depth interviews was used to gather data on the illegal wild meat supply chain and to create a sustainable wild meat trade framework. The interviews were conducted in English, Nyanja, or a combination of both. In cases where the interviewee was not fluent in English, a Nyanja field assistant acted as an interpreter.

### 2.3. Participant selection

The purposive sampling method [49], followed by the snowballing sampling method [50], was used to engage participants in data collection. The key informants were purposefully selected on the basis of their knowledge of illegal game and the game meat trade. The inclusion criterion was individuals with professional or operational knowledge of the illegal wild meat trade; the exclusion criterion included a lack of relevant expertise or refusal to provide informed consent. Snowballing sampling was repeated until the data saturation point was reached. In-depth interviews were conducted with 38 key informants. The interviewed individuals were selected mainly from key organizations that are responsible for regulation, control, and wildlife conservation. The interviews included those with government officers, country directors and focal points, project managers, consultancies, academicians, researchers, and butchers Table 1.

**Table 1:** Key informants and the supply chain stage about which they were knowledgeable.

Key informant	Number	Stage
Law enforcement	6	Entire chain
Conservationist	16	Entire chain
Research Organizations	3	Entire chain
Regulators	4	Entire chain
Academician/researcher/consultancies	4	Entire chain
Butcher	2	Distribution and selling
Ranger	3	Sourcing and distribution

### 2.4. Data collection and transcription

The interviews were structured in three sections: general thoughts on the wild meat trade in Zambia, understanding the wild meat supply chain, and sources and destinations of wild meat. The interviews ranged between 25 minutes and 1 hour 40 minutes and took place in offices and over telephone calls. In-person interviews were recorded using a recording device. Phone interviews were recorded. Zoom/Microsoft and Google Meet interviews were recorded on a computer. In all these interviews, consent was sought first. Interviews that were conducted in Nyanja were translated to English before they were transcribed . All the interviews were then transferred to a computer for transcription. Artificial intelligence (AI) Whisper [51], which is a function that is embedded in the Python application, was used for data transcription. The recorded audios were input into the application, and Microsoft text transcripts were the output. The interviews were transcribed in English. To ensure transcription quality, all of the Whisper-generated transcripts were manually reviewed. The review focused on key elements such as completeness, accuracy of terminology, and contextual consistency. Any discrepancies were corrected manually. The output transcripts were then uploaded to Nvivo 12 for coding (data analysis).

### 2.5. Thematic content analysis

Grounded theory was used for coding as previously described by Goodall et al. [52] and Milstein et al. [53]. Grounded theory uses the inductive method of coding [54], which has the advantage of reducing study bias [55]. This is because inductive analysis primarily uses detailed readings of raw data to derive concepts, themes, or a model through interpretations made from the raw data by a researcher without the restraints imposed by structured methodologies or preconceptions in the data collection and data analysis procedures imposed by investigators [54]. This approach is used when not much is known regarding the phenomena being studied [56]. As the foundation of grounded theory, concurrent data collection and analysis were performed [57]. Constant comparative analysis using the iterative process was used for data analysis [47]. Coding was performed in three phases: initial coding, which fractured the transcripts. Intermediate coding, which weaved the fractured data back together again into an organized theory and advanced, which integrated the final grounded theory [48,47].

### 2.6. Distance from national parks (NPs) to Lusaka

The proximity of wild meat to end markets, whether short- or long-chain, may increase or decrease food safety risks [58]. Hence, the distance from the NPs to Lusaka city by was determined. Google search was used to determine the distance.

### 2.7. Ethical clearance and participant consent

Ethical clearance was obtained from the Tropical Disease Research Centre (TDRC/124/09/23). All participants were informed of the study objectives through a written consent form, which they read and signed prior to participation. To ensure anonymity, no names or personal identifiers were recorded on the data collection tools. Instead, unique codes were used for each participant. All the completed consent forms and data were securely stored in locked cabinets (for physical forms) and password-protected digital files (for electronic data). Access was restricted to authorized members of the research team only. The researchers faced one ethical challenge with one organization, whereby they had to pay for a research permit to that organization to interview their staff, and after the payment they were not given access to the staff, so they had to interview other suggested organizations by informants.

## 3. Results and discussion

### 3.1. Description of the supply chain

To illustrate potential sources and pathways of zoonotic transmission, a description of the typical processes that occur from forest to folk were described.

#### 3.1.1. Sourcing and processing.

Poachers are residents of the constituencies surrounding game management areas (GMAs), national parks, or general protected areas. They are supplied with illegal firearms by people from urban areas, and in return, they bring them wild meat. They are also supplied with clothes, money, or any other items that are valuable in the community that they can use to sustain the lives of their families. As reported by this informant:

*“So these individuals, that do poaching, get help from the traders or dealers that will come from the city. The poachers are supplied with illegal firearms and ammunition. They are also supplied with clothes, money, food, and school children’s needs, such as uniforms or books or other items that are valuable in the community, and in return, they will supply them with wild meat.*

This is not the first study to report the provision of hunting equipment and valuables to poachers. Eniang et al. [59] revealed the rise of contract hunting as a result of stiff competition in south-eastern Nigeria. This is the practice of providing either bulk cash upfront or hunting inputs (shotgun, cartridges, carbide, headlamps, and machetes) to the hunter by an urban dealer who, in return, provides the wild meat to the dealer. It was reported that the poachers enter a protected area, usually a wildlife-dense area, which can be a GMA or NP. They do this with ease since they are very familiar with the protected zone/s. They know how to get around dangerous animals. In some circumstances, they form poachers’ camps.

For hunting, they use either guns or wire snares. As reported by one of the informants here:

*“They would either use guns or wire snares. The advantage with the guns is that they can identify an animal and kill it immediately. They are relatively inexpensive to make the only challenge they pose is that they do require some skill to make sure that the shotgun does not harm the poacher, additionally they make noise and they might attract law enforcement. Some use wire snares because they are silent and they can be spread in a relatively small area and have a higher chance of success, unlike with the shotgun where the poacher might miss.”*

Another informant reported that:

*“Mostly, they use firearms, but a few are using snares now because the poachers are getting quite sophisticated. They also know a gunshot sound can reach very far, which can prompt officers to go in their direction and try to apprehend them”.*

The use of shotguns reported by the informants was previously reported by Lindsey et al. [9] in South Luangwa and Kafue NP, Zambia; Overton et al. [16] in the Greater Kafue ecosystem in Zambia; Mendelson et al. [60] in Takoradi Ghana; Van Vliet et al. [61] in Yangambi, Democratic Republic of Congo (DRC); Milstein et al. [53] in Guyana South America; Van Vliet et al. [62] in the coastal area of Guyana; Akpan et al. [20] in Lagos Nigeria; and Masudi et al. [45] in the Nairobi Metropolitan Area, Kenya.

It was reported that most of the guns that are used are muzzle-loading homemade shotguns; in some circumstances, deformed bullets are used. The types of firearms reported are likely to increase the risk of falling ill from a zoonotic disease after butchering wild meat due to construction and impact. This is a result of the impact, which causes damage to the animal and increases the likelihood of microbial contamination. Ammunition construction, the impact energy of the ammunition, and improper shooting accuracy influence the initial microbial load [63]. The use of deformed bullets caused higher initial levels of *Enterobacteriaceae* and *E. coli* in a study performed by Korkmaz et al. [64]. It was reported that since poachers might not be trained hunters, they might not deliver a single kill shot, allowing escape distance before the animal dies, which is a risk factor. A wounded animal may shed pathogens along its escape route through bleeding or by spreading bodily fluids, which can contaminate the environment or other animals, increasing opportunities for indirect transmission.

The use of snares was also reported by Overton et al. [16] in the Greater Kafue ecosystem and by Lindsey et al. [9] in South Luangwa and Kafue NP both in Zambia, Mendelson et al. [60] in Takoradi Ghana and Van Vliet et al. [62] in Yangambi, DRC. It was reported that the danger of snares is that they can constrict the animal until the animal stops breathing. When the animal falls down and eventually dies, it can take a day to three days for poachers to visit and check the snares. If the animal has died, perhaps two to three days before the poachers return to check the snares, they may find the animal bloated. When snares are used, swelling of the intestines increases the probability of the gut being damaged during its removal; in addition, snares are also likely to be contaminated [65]. A study by Lindsey et al. [9] in South Luangwa and Kafue NP, Eniang et al. [59] in Oban Hills, Nigeria, and Van Vliet et al. [62] in the coastal area of Guyana reported the use of dogs during hunting. This tendency was also reported in this study. The use of bows and arrows previously reported by Milsten et al. [53] in Guyana, South America, and Van Vliet et al. [62] in the coastal area of Guyana were not reported. Furthermore, this study did not report the use of traps reported by Van Vliet et al. [62] in the coastal area of Guyana or Akpan et al. [20] in Lagos, Nigeria. Whether the animal has been killed by a wire snare or a shotgun. The animal is usually moved outside of the protected area, as highlighted by this informant:

*“The skinning, removal of waste and internal organs, and smoking happen mostly in the neighboring bushes near the NPs, depending on whether there is law enforcement or not in the NP. If there is law enforcement, they might have to drag it or carry it for a few kilometers to a safer area where they would process it.”*

Dragging the animal after killing was identified as a high-risk factor [64]. During dragging, the fur of the carcass might be contaminated with soil or bacteria, which could be transferred to the meat during evisceration or skinning [64]. Casoli et al. [66] highlighted that bacterial contamination of carcass fur is a major source of cross-contamination on the meat surface. Depending on the number of animals they need, they may stack them and then process them once. This practice increases the duration between killing and evisceration, which is a risk factor [64,67,68]. The longer the delay between killing and evisceration is, the higher the risk of zoonotic pathogen transmission due to bacterial proliferation, gut rupture, and environmental contamination. Immediate and hygienic evisceration is a critical control point in reducing zoonotic disease risks in wild meat processing [69,70,71].

When they reach the processing point, they let the blood out and skin it on the ground, as they do not have time to hang it properly. Processing wild animals in the field increases the risk of falling ill from a zoonotic disease after butchering wild meat, which is due to poor hygiene and sanitation [72]. Sometimes, skinning is done using rusty knives, and in some circumstances, it is done hurriedly and without proper lighting. All these factors increase occupational health hazard risks, which increases by so doing increase the probability of a spillover. A study by Subramanian [73] reported that 38% of respondents cut themselves regularly during wild meat butchering. LeBreton et al. [74] reported that only 2% of people said they took precautions against contact with animal blood and fluids while butchering wild meat. LeBreton et al. [74] and Van Vliet et al. [75] reported that the risk of zoonotic disease transmission is highest at the butchering stage, e.g., during skinning, opening of the body cavity, removal of organs, and cutting of meat. Simian immunodeficiency virus and human immunodeficiency virus type 2 sequences derived from animals and humans from the same immediate geographical area were found to be most closely related, which implicated hunting [76]. Wolfe et al. [77] reported similar retroviral zoonosis in people who had direct contact with fresh nonhuman primate wild meat and concluded that transmission was a result of a combination of urban demand for wild meat and greater access to primate habitats provided by logging roads. Kurpiers et al. [24] highlighted that, considering that in poaching, the animals are not inspected, sick animals may be less successful in evading hunters and hence more easily hunted, thereby increasing the risk of disease transmission to hunters.

After skinning, they remove the offal. There is no washing. After removing the offal, they cut the carcass into pieces and smoke-dried them. In some circumstances, processing and smoking are performed within an NP or GMA depending on the security, as already highlighted. The practice of processing wild meat in the forest is in agreement with what Overton et al. [16] reported in a study that was performed in the Greater Kafue ecosystem, Zambia. This, however, differs from findings in Guyana, where wild meat is butchered in the village Milstein et al. [53].

The smoking is done by fire in a centralized place or a camp where they put drying racks; they set a very large fire and smoke it; sometimes it is smoked with salt; sometimes they just leave it to dry naturally, and sometimes they do not smoke at all depending on the season, as reported by this informant:

“*What happens is hunting happens year round, and you find that sometimes, depending on the season, if it is not smoked, it might even start to spoil because of exposure of all the microbes in the environment”.*

Poaching was reported to be performed year round, with the supply affected during the rainy season due to difficulty accessing some of the NPs. A similar phenomenon was reported by Lindsey et al. [78], who noted a peak in illegal hunting during the late dry season and a decrease during the rainy season in the Save Valley Conservancy, Zimbabwe. In Nairobi, Kenya Masudi et al. [45] reported that the poaching, sale, and consumption of wild meat differed across seasons, with hunting peaks occurring during the late dry season. The practice of hunting during the rainy season increases the risk of falling ill from a zoonotic disease after butchering wild meat due to contamination.

The camping of hunters and smoking of wild meat reported in this study were also reported by Van Vliet et al. [75]. Smoking is performed for preservation, and in addition, it makes the meat lighter for easy transportation. The practice of smoking and drying wild meat in bush camps was also reported by Overton et al. [16]. Drying meat reduces the risk of falling ill from a zoonotic disease after processing wild meat, but owing to unhygienic handling, the meat may become contaminated [79].

After smoking, the meat is bundled. Bundling is the process of packaging meat to sell it. The bundling is done in such a way that when it is packed, it is sold in a standard size bundle that every poacher would use. The bundling was reported to be performed under unhygienic conditions. Katani et al. [80] reported that illegal meat processes are conducted under unhygienic conditions, which affects the microbiota profile of the meat as well.

It was revealed that females are not involved in hunting, which is in agreement with what was reported by Van Vliet et al. [75] in Yangambi and Kisangani in the Democratic Republic of Congo (DRC) and Akpan et al. [20] in Lagos, Nigeria. The same studies also reported female actor involvement in processing, which was not reported in this study. In the Lusaka supply chain, this means that women are at a lower risk of exposure than in the mentioned studies, considering that the highest risk of exposure in the illegal wild meat supply chain occurs during butchering [73].

#### 3.1.2. Transportation and distribution.

After they have smoked it, meat is then transported in plastic bags and sacks to the village. One of the key informants highlighted that:

*“Once it is smoked and they have got enough, they pack it in sacks, then they will start transporting it, using foot and bicycles from the bush to the villages and hand it over to the traders who would take it to the city, to the ones that would have provided them with goods and ammunition”.*

Still regarding transportation, another key informant reported that:

*“I have seen people using scotch carts, bicycles, vehicles, and some people use foot, especially in very difficult terrain where vehicles cannot reach. Those near rivers use canoes; these are different modes of transport available”.*

The packaging material revealed by the informants was also reported by Overton et al. [16]. These packaging practices lead to meat contamination and increased risks, even if the meat is dry. The same modes of transport were reported in a study by Borato and Gore [81] in Pointe Noire, DRC. The poachers also help the traders enter the city, in most cases, without being apprehended on the way. If the poachers did not come through the villages, the meat would be taken to the area where it would be sold directly. It was reported that people poaching wild animals are not people selling wild meat in urban areas. There are middlemen or people who are based in Lusaka who travel to one of the protected areas or travel to the village where the middlemen source or purchase the smoked dried wild meat and then smuggle it to the city. As reported by this informant:

*“Often, in this business, the people poaching the animals are not the people selling the meat in urban areas; usually, there is a middleman or dealers, so those people who are based in the city would travel to one of these protected areas or travel to the village where the poachers are, purchase the smoked dried meat, and then smuggle it into the city”.*

The distribution of illegal wild meat through middlemen or intermediaries was also reported by Katani et al. [80] in the Serengeti ecosystem in Tanzania and Eniang et al. [59] Oban Hills Nigeria, Lattine et al. [82] Suluwesi Indonesia and Van Vliet et al. [61] in the Yangambi DRC. It was reported that the meat is carried by private vehicles or public transportation, such as buses; they may hide it in other products, such as bags of maize or bags of other products, sometimes in their luggage, for example, in suitcases, in any way that can disguise it so that the smell is not easily detected. A study by Latinne et al. [82] reported that drivers shuttled back and forth between North Sulawesi and other provinces to collect wild meat from local dealers or hunters; this practice differs from the hiding of wild meat in public and private transport reported in this study. In areas where fishing is done the poachers put the meat under the fish in some circumstances. It was revealed that they use anything that can cover the smell of meat so that it is not detected. Hiding wild meat in other products, such as bags of maize or bags of other products, such as fish, can lead to cross-contamination. The practice of covering the smell was also reported by Mumba et al. [23]; in Mumbwa, Zambia, the study reported illegal wild meat traders make bundles that fit into sacks, apply perfumes to sacks, and smuggle. The practice of hiding meat in agricultural produce was also reported by Borato and Gore [81] in Pointe Noire, Republic of the Congo. The practice of hiding meat in personal luggage as a way to disguise it so that the smell is not easily detected also increases microbial contamination. Van Vliet et al. [75] highlighted that cross-contamination of wild meat is highest during transportation because of rain, dust, flies, and other foods.

### 3.2. Species

These were reported as the five most preferred and consumed species: buffalo (*Syncerus caffer*), kudu (*Tragelaphus strepsiceros*), impala (*Aepyceros melampus*), warthog (*Phacochoerus africanus*), and hippopotamus (*Hippopotamus amphibious*) Table 2. Buffalo is the most preferred species because of its close resemblance to cattle and its abundance in many protected areas. The other species were preferred because of their taste.

**Table 2:** Reported sources of illegal game meat, hunted species and distance to market.

Source	Species	Distance to Lusaka (km)
Kafue N.P	Buffalo, warthog, duiker, bushbuck	348
Lochinvar N.P	Wildebeest, zebra, kudu, bushbuck	229
Lower Zambezi N.P	Buffalo, wildebeest, antelopes, hippopotamus	179
Blue Lagoon	Buffalo, kafue lechwe, zebra	120
North Luangwa N.P	Buffalo, wildebeest, antelopes, hippopotamus	773
South Luangwa N.P	Buffalo, wildebeest, antelopes, hippopotamus	559

As reported by this informant:

*“First, it is because of their availability, like the buffalo; second, it is also the taste, people like the taste. With respect to taste, the first preference is the warthog, followed by the hippopotamus. The warthog has certain unique taste characteristics. Hippopotamus meat also has very unique characteristics. So you find that seasoned wild meat consumers will either go for a hippopotamus and warthog if these are not available, they will ask for a buffalo”.*

Regarding what determines the species that are most hunted, some participants said it depends on the locality and what is available there. For example, in Liuwa NP, in the western part of Zambia, the most common animals are wildebeest and zebra, and most of the carcasses and dried meat that are recovered by authorities consists of wildebeest and zebra meat. It was reported that other animals might be poached if they come across them, but usually, poachers would target something readily available in that location. In locations where there are different species, such as Luangwa and Kafue NP, they kill anything indiscriminately without really having to choose. The Kafue flats and the Bangweulu wetlands are specialized N.P.; most of the abundant animal species are kafue and black lechwe, respectively, and so they poach lechwes more than any other species in these areas. It was reported that recoveries that have been reported in Kafue NP were primarily sable and reedbuck, and these are the antelope species that are closer to community areas where they are likely to be poached. Another participant mentioned that wild meat is area-specific according to what is available in a particular area and how easy it is to catch that wildlife. The informants reported that the most targeted wildlife species are larger antelopes and buffaloes; consequently, logistically, it is more productive to shoot a large animal and leave the protected area than to shoot small antelopes, in addition, larger animals have more meat. Taking into consideration that the same effort and risk to poach an impala is almost the same effort to go and poach a buffalo; hence, they would rather poach a buffalo.

This study revealed the hunting of ungulates. The hunting of ungulates was previously reported by Eniang et al. [59] in Oban Hills, Nigeria; Van Vliet et al. [61] in Yangambi, DRC; Van Vliet et al. [62] in the coastal area of Guyana; Akpan et al. [20] in Lagos, Nigeria; and Masudi et al. [45] in the Nairobi metropolitan area of Kenya. Fa et al. [83] reported that ungulates were the most consumed species in Nigeria, followed by primates. Taylor et al. [84] highlighted that in terms of the biomass offtake, ungulates are generally the most prominent group. This also agrees with the findings of Staal et al. [7], who reported that ungulates, such as antelopes, tend to be the most frequently hunted animals and the most important in terms of overall biomass extracted. Woolhouse and Gowtage-Sequeria [85] reported that of 816 human pathogen species known to be zoonotic, the most important hosts in terms of the diversity of zoonotic pathogens supported were ungulates, followed by carnivores, rodents, non-mammalian species, primates, other mammals, and then bats. Bats and non-human primates are the most common species among those documented as leading to confirmed zoonotic disease in people, followed by wild rodents and, less frequently, buffalo and wild pigs (ungulates) [86]. In determining which mammal groups pose the greatest zoonotic risk to humans, Han et al. [87] highlighted that rodents and carnivores have high zoonotic potential compared with ungulates. This means that ungulates are lower-risk species. However, Kock and Caceres-Escobar [3] emphasized that without human case data and confirmatory diagnostics on zoonotic and emerging infectious disease pathogens transmitted or derived from wildlife species, it is not possible to determine with certainty the importance or risk of these hosts and reservoirs. This means that it is difficult to know without data the risk posed by the ungulates that are being illegally traded in Zambia, considering that most species have not been studied. Cooney et al. [88] stated that for most species, there is a need to increase the understanding of the risks presented by their trade to improve the management of impacts on livelihoods. This is important considering the high variability of markets, actors, and factors that drives the trade throughout the wildlife trade value chain.

### 3.3. Distance from source to market

The proximity of wild meat to end markets, whether short- or long-chain, may increase or decrease food safety risks [58]. A short chain is defined as a food chain that is less than 80 km long [89]. The following areas were mentioned as the major sources; their respective distances from Lusaka were also estimated: Kafue NP (348 km), South Luangwa (559 km), North Luangwa (773 km), Lochinvar NP (229 km), Blue Lagoon NP (120 km), Lower Zambezi NP (179 km), and Kafue Flats (46 km) Table 2. All the other distances except Kafue flats represent long chains. Wild meat is transported over this distance in the absence of a cold chain, increasing the risk of falling ill from a zoonotic disease after its transportation [90,91]. A cold chain is a system that keeps meat at the right temperature to prevent spoilage and to ensure food safety [92]. The short- and long-chain sourcing of wild meat was also reported by Reuter et al. [93], with wild meat being sourced from 10–700 km from the source. Van Vliet et al. [75] described the wild meat trade in Yangambi, DRC, as having a relatively short chain. Several reasons were mentioned as to why the reported areas are the most poached, including the size of the NPs, animal diversity, low security, easy access, and good road networks to some of the national parks.

### 3.4. Market

It was reported that there is a steady market for wild meat in Lusaka for household consumption. There are also many restaurants that have wild meat on their menu. Therefore, there is always a market, either for household consumption or for business. Large population, high incomes, and easy transportation on both roads and rails were noted as reasons why Lusaka is the major market for wild meat.

### 3.5. Sales

Once the wild meat reaches Lusaka, it is stored if the middleman has refrigerators; otherwise, it is stored in sacks or plastic bags. The meat is sold directly to customers whom they know. The selling of the meat directly without any market place/s reported in this study differs from what has been previously reported, market stalls and chopbars in Takoradi Ghana Mendelson et al. [60] wild meat markets, in Oban Hills Nigeria Eniang et al. [59], open markets and supermarkets in Suluwesi Indonesia Latinne et al. [82], wholesale, open markets and food stalls in Yangambi DRC Van Vliet et al. [61], wholesales in Lagos Nigeria Akpan et al. [20], retail and butcheries in the Nairobi Metropolitan Area, Kenya Masudi et al. [45]. The difference indicates the sensitivity of selling illegal wild meat in Lusaka, Zambia. The reasons behind the freedom of selling in the other supply chains might be as follows: the regulations allow wild meat hunting and trading; the regulations present contradictions or gaps Van Vliet et al. [61] or the regulations are there, but there is poor enforcement, and people can still trade illegally without consequences. Eniang et al. [59] noted that in Oban Hills, Nigeria traders were aware of the illegality of their trade yet economically compelled to continue.

Selling was reported to be done by both men and women. This practice was also reported by Akpan et al. [20] in Lagos, Nigeria, who reported that female actors generally outnumbered males in the value chain, with retail nodes specifically being female dominated in Lagos, Nigeria. Olunusi [43] also reported that female actors dominated selling in Ibadan Metropolis, Nigeria. Hence, conservation efforts and zoonotic risk prevention strategies should be gender inclusive Akpan et al. [20].

The importance of relationships in the illegal wild meat trade reported was also highlighted by Boratto and Gore [81] in Pointe Noire DRC. During these distributions, no hygienic protocols are observed, increasing the chances of contamination. It was reported that many traders go to offices because they prefer to sell wild meat to people who are well-off because they can sell it on credit and then come back and collect the money later. Since it is illegal, they do not want to spend much of their time with it; hence, they prefer to sell it as soon as possible. The practice of selling game meat to restaurants, offices, and households was also reported by Borato and Gore [81] in Pointe Noire DRC. This practice increases the risk of falling ill from a zoonotic disease after selling wild meat, considering that handling and selling are unhygienic. The practice of not storing wild meat in the fridge, especially when it is dry and, in some circumstances, when it is fresh, increases the risk due to the lack of a cold chain [94]. It was reported that more illegal wild meat is traded than legal game meat. As reported by this informant:

*“From what I can tell, the problem is there is limited legal game meat on the market. Hence, the demand far outweighs the supply, that is why we have a flourishing illegal game meat market, so there is more of the illegal game meat on the market than the legal”.*

What was reported by this informant is in agreement with what was also reported by Overton et al. [16] and FAO et al. [95].

### 3.6. Consumption

It was reported that consumers may think they are purchasing certain species, but what they are purchasing may be something different. What consumers think they are purchasing would probably be kudu, buffalo, and impala, but what they are consuming can be something else. An instance was reported by an informant:

*“Hippopotamuses, which were dying of anthrax, were not marketed as hippos but as buffalo meat. Sellers would lie to customers that they were buying buffalo. This is mostly because people love buffalo meat, and it is the reason why it is among the most trafficked and fraudulently sold wild animal meat.”*

The consumption of meat from sick animals is a direct way to transmit disease from animals to humans. In 2023, twenty-six people developed sores on their faces, arms, and fingers after consuming meat from three wild hippopotamus carcasses infected with anthrax in the Sinazongwe district of southern Zambia Province [96]. This is an indication that poor handling and hygienic standards are not the only zoonotic vectors. Another example is the direct transmission of *Mycobacterium bovis* [90] as a result of consuming undercooked meat.

The issue of fraud was also highlighted by one of the key informants:

*“We did a research and when we sequenced wild meat which was sold as impala meat, it was not even impala meat, it was a baboon. I am told they even mix wild meat with dog meat, so that is the danger of eating illegal game meat you can eat anything.”*

It was reported that animals found dead in the forest are also skinned, dried, and mixed with hunted dried meat to make bundles and sell to people. The consumption of dead animal meat increases the risk of falling ill from a zoonotic disease. When an animal dies, it starts to decompose, and microbial succession starts. Microbial community succession continues until the carcass ruptures due to the presence of commensal microbes, air, and insects [91]. In the case where pathogenic microbes such as *Bacillus anthricis* and *Myobacterium bovis* are present, a succession of pathogenic microbes also commences. If an animal is not infected by any pathogenic microbes, the following ways may lead to carcass contamination with pathogenic microorganisms: insects, air, vultures, and carnivores, which may scavenge part of the carcass, wounds, and injuries [97,98]. The risk of falling ill from a zoonotic disease after consuming wild meat increases as the number of days’ increases due to microbial succession. This succession may lead to spore formation from bacteria such as *Bacillus anthracis,* which are difficult to kill during cooking or grilling, increasing the risk. Snared animal meat risks are greater than those of shot animals and lower than those of dead animals. However, this varies depending on whether the snare caused any injuries [98] and whether the hunter arrived while the animal was still alive or dead. If the hunter arrives when the animal is already dead, the factors discussed related to dead animals also affect the meat. Snares also have an additional risk of catching sick animals, which further increases the risk of falling ill from a zoonotic disease after consuming wild meat. Additionally, there are animals that escape the snares but become injured and die from infection [99], which also poses a greater risk.

The misrepresentation of meat species, as revealed by this study, is a risk. The risk is even greater if the species are high-risk species, for instance, primates [86], or those from a high-risk zone, which is a geographic area where the likelihood of zoonotic disease spillover is significantly increased due to various environmental, ecological, and human behavior-related factors [100]. Duporge et al. [101] highlighted that household surveys and illegal wild meat market surveys often experience the same problem where consumers or sellers do not know the species they are consuming. In a study done by Schilling et al. [102] in the Serengeti ecosystem, 30% of the sellers misreported wild meat species.

In regard to the method of preparation, the most common method that was reported was braising and boiling, which reduce the risk of falling ill after consumption of wild meat [81,103].

### 3.7. Sustainable wild meat trade framework

**Fig 1:**
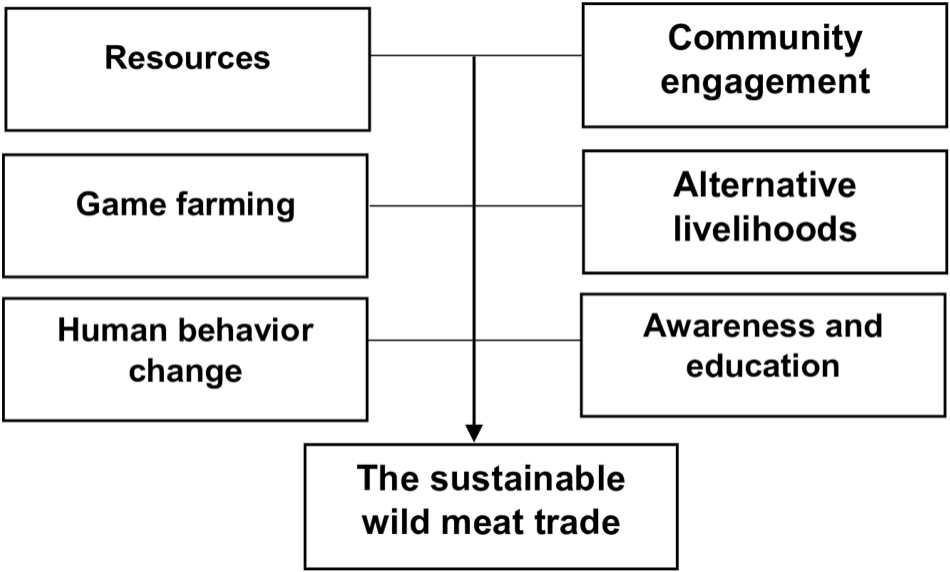
Proposed sustainable wild meat trade framework in Zambia.

Informants reported challenges and made suggestions regarding the illegal wild meat supply chain, which were used to develop a “sustainable wild meat trade framework” Fig 1. in Zambia. Considering the socioeconomic needs of human populations, the framework can result in the sustainable management of wild animals to maintain their populations and habitats over time. The framework is explained below Fig 1.

One of the elements needed for sustainable legal wild meat trade is regulations that regulate the hunting and trading of wild meat. Regulations are not included in the framework in Fig 1 because they already exist. As reported by this informant:

*“We have all the laws in place. The challenge is implementation; you find that there is a law that regulates illegal hunting, but there are no people who are monitoring illegal hunting. They are not there on the ground.”*

Holmern et al. [104] highlighted that law enforcement is crucial for curbing unsustainable and illegal exploitation of animal populations. Stricter law enforcement can also help create awareness among consumers [105]. Da Silva and Bernard [106] stated that, similar to any other developing nation, **enforcement** through the penalization of illegal wildlife traders is something that the legal system needs to commit to. Regulations and enforcement to address illegal wildlife trade in general are not considered a priority by entities responsible for enforcement agencies [1]. As a result, high penalties have not yet been applied in many countries [107] Zambia included, and stiffer penalties may provide stronger disincentives to engage in the illegal wildlife trade. The government also needs to have deliberate policies that benefit the local people, as highlighted by this informant:

*“We should not run away from the fact that the wildlife is being hunted and that wild meat is being consumed in the country, so maybe there should be deliberate policies that should be put in place to allow people to access wild meat, of course with licenses at affordable prices for the local people. We know that there is trophy hunting, but most local people cannot afford it, that is the reason why it is mostly done by foreigners. For locals, authorities should reduce the license cost. I remember buying an animal during the hunting period and the cost was high, so if the cost is reduced, illegal hunting will be discouraged.”*

**Resources** for law enforcement, judiciary, and other entities entrusted with stopping illegal behavior and prosecuting offenders should include funds for staff, supplies, training, enforcement, and judicial operations. In addition, they ought to be held to the highest standards of honesty and be granted sufficient legal authority to use all available investigative methods in the battle against corruption. Law enforcement personnel need to be compensated fairly to prevent corruption [108]. As reported by one of the informants:

*“Funding is the main challenge; hence, employment levels in DNPW are low, community officers are few, and because the same people are underpaid, the officers end up poaching themselves”.*

Governance of the wild meat trade could be made more effective by ensuring that law enforcement agencies have the human resources, legitimate authority, and financial resources needed to enforce national laws and tackle corruption [10,109]. This is not the case because of the under appreciation of the severity of illegal hunting in many African countries; as a result, protected areas do not have sufficient resources, making it a challenge to make conservation efforts successful [78]. ‘T Sas-Rolfes et al. [110] noted that wildlife resources are not properly managed principally because of a lack of resources for protected area management, thereby impeding effective law enforcement. As a result disabling the wildlife trade by relying on government institutions and their financial and administrative resources seems impossible [111] in many instances. Owing to low resource allocation in developing countries, illegal hunting is likely to continue [10]; hence, the government and other private and public organizations should invest more in law enforcement resources both human and financial.

Local communities and consumers can help reduce the illicit wild meat trade through **community engagement** by offering additional help for wildlife management [94,112]. They must also be well versed in local regulations concerning the trading of wildlife, the dangers associated with illicit commerce, and the preservation of species. Prioritizing justice and diversity is necessary to stop illegal trade and develop new conservation approaches. This entails adopting conservation tactics directed by indigenous people and respecting land rights [113]. One informant reported:

*“The policies have to benefit the local people who are living next to the resources because if they are frustrated, they will also frustrate the government efforts in the conservation of wildlife resources.”*

To provide different species with acceptable, sustainable, and legal alternatives to wild wildlife supplies, some have pushed for **game farming**, [114]. These actions could lead to a decline in illicit trade [1]. To fully pursue the possibility of game farming, increasing public knowledge can help prevent unsustainable increases in hunting in wild species [115]. Educational activities for game farming are needed [1,106]. Selectively bred variants on farms can also lead to a greater desire for commercially produced species that have appealing characteristics or health benefits, which may be crucial for attracting consumers who otherwise perceive captive-bred specimens as having lower value than their wild counterparts do [1]. In a previous study, Marescotti et al. [116] revealed that hunters preferred hunted products over farmed meat. Fantechi et al. [117] highlighted that consumers tend to prefer wild game since they perceive it as more natural. Hence, it might be challenging to convince wild game consumers to change to farmed game, particularly seasoned wild meat lovers. Regulations about captive breeding and propagation might need to be relaxed to encourage a profitable endeavor that helps the local economy and wildlife [1,105].

According to Can et al. [19], prohibitions are unlikely to eliminate illegal wildlife trade and associated public health risks. This is because such policy decisions have the potential to encourage illegal wildlife trade activity and can negate the values of human equity and sustainable development [114]. Unless they are backed by related **human behavior, change** initiatives aimed at lowering the involvement and dependency of those involved in both purchase and sale [19]. Government officials and specialists must spearhead initiatives to modify human behavior by utilizing best practices from social marketing, psychology, economics, and behavioral science [39].

Putting measures regarding the use of local wild meat while ignoring broader rural development policies that allow indigenous peoples and local communities to pursue alternative means of subsistence can exacerbate the already existing rift between communities and conservation authorities [118]. Because these communities frequently rely on wildlife for food and making a living, preventative efforts such as supporting **alternative livelihoods** (e.g., agroforestry, livestock, sustainable agriculture, ecotourism) should focus on reducing reliance on commercial wildlife markets and trading. These are preventative because they address the root causes (poverty, lack of options) that lead communities to depend on wild meat. To reduce reliance on wildlife markets and trade for revenue, local people and impoverished people in rural areas need to be the focus of alternative livelihood programs [1,12]. This informant reported that:

*“There is a lot of demand for wild meat in urban areas because it is considered to be tasty, to be a healthy alternative to beef and other protein sources, and because of the high demand. It is sold at a premium price because of the demand and premium price at the end of the day they are people in rural communities, many of whom do not have alternative sources of income who turn to poaching as a quick way or a relatively easy way to make money”.*

Another highlighted that:

*“There are a few wildlife opportunities that are created for the indigenous people. You find that it is very easy for people with funds to influence the local communities to become involved in illegal wild meat hunting.”*

Partnerships between local governments and communities are crucial to providing incentives for robust and persistent income streams that are independent of wildlife markets and trade [40,108].

**Education** could concentrate on pertinent legislation controlling the trade and exploitation of wildlife and the detrimental effects of commerce, such as animal welfare and zoonotic illnesses [119]. For any intervention to succeed, social and cultural elements need to be taken into account [120]; hence, both of these factors need to be considered when carrying out awareness and education campaigns. In other situations, although it is often lacking, political support is required for campaigns. Planning with local communities and stakeholders in mind is essential to maximize chances of success and efficacy and avoid the waste of resources, or worse, adverse social effects [1]. Social media can offer useful information about consumption trends and, by raising awareness and educating people, help alter attitudes toward illegal trade [121]. The communities also need to be educated on the importance of wildlife, as reported by this informant:

*“The fact is they are people mostly in rural communities or living next to protected areas who are selling wild meat as a way to make money these people do not understand the value of wildlife and other resources outside of tourism.”*

Hilderink and De Winter et al. [122] argued that conservation education programs are unlikely to influence hunting due to the cultural and religious connotations of wild meat; alternatively, they suggested health-risk education, which they noted has the potential to minimize hunting rates since it can impact the social acceptability and demand for wildlife products. Knapp [123] suggested that in areas where high educational costs are causing poachers to hunt, the creation of scholarship funds or price reductions for secondary education might be more advantageous than increasing anti-poaching patrols. The provision of scholarships can be a long-term solution because, in many cases, wild meat hunters are unemployed and have little education [9]. Importantly, the success of such efforts also depends on hunting reasons, where poachers hunt because it is part of their cultural heritage, scholarship funds or any other form of monetary assistance are unlikely to reduce poaching [123].

Some approaches have also been suggested to address the illegal wildlife trade. Fukushima et al. [1] proposed tools that can be used to address the wildlife trade chain through a wildlife circle trade chain. Hu et al. [124] proposed a theory of change for reducing illegal wildlife-based medicine consumption. Even though these are not frameworks, they present ways to address the illegal wildlife trade. The main difference between the referenced approaches and the one proposed in this study is the inclusion of bans in [1]. We agree with Booth et al. [125] on the impact of bans on food security, taking into consideration that more than 700,000 tons of legal meat in Zambia are consumed in Zambia [95]. Hu et al. [124] focused much on behavior change, which is imperative, as already discussed, but in the case of Zambia, there is a need for more. In summary, what also differentiates our approach is that it is formulated from in-depth interviews.

### 3.8. Summary of the identified themes

The actors in the **sourcing** stage are poachers from communities near NP and GMAs. They are driven by poverty, a lack of alternative livelihoods, and the provision of supplies (e.g., firearms, clothes, cash) by urban traders. Hunting methods include the use of homemade shotguns, wire snares, and dogs; snares pose a zoonotic risk because of delayed carcass retrieval. The species targeted are primarily ungulates such as buffalo, kudu, impala, warthog, and hippo, which are chosen for taste, size, and availability.

**Processing** involves several practices. These include skinning, evisceration, and smoking in forests or bush camps, often without hygiene or proper tools. The associated risks are as a result of the use of rusty knives, poor lighting, contact with blood and fluids, and exposure to contaminated soil, which increase the risk of zoonotic transmission. The time lag between killing and evisceration contributes to microbial proliferation.

**Transportation** modes include foot, bicycle, canoes, scotch carts, public transport, and private vehicles. With respect to packaging, meat is carried in plastic bags or sacks and is hidden in other products to avoid detection. Potential risk factors include cross-contamination from fish, dust, or other goods and no cold chain during transport.

The main actors in the **distribution and sales** include middlemen and urban traders; poachers rarely sell directly. Sales methods include direct sales to known customers, sometimes in offices or homes; no open markets due to illegality. Women are more involved in selling than hunting.

The potential consumer risks associated with **consumption** are a result of species misrepresentation (e.g., hippopotamus sold as buffalo) and the consumption of meat from dead or diseased animals. The common preparation methods involve boiling and braising, which reduce but do not eliminate pathogen risk.

The proposed **sustainable framework** is composed of the following components: community engagement, game farming, alternative livelihoods, awareness and education, and human behavior change. The goal of the framework is to promote safe, legal, and sustainable wild meat trade while reducing zoonotic risk.

## 4. Recommendations: Operationalization of the framework

The proposed framework in Fig 1 is conceptual; to operationalize the framework, the involvement of all the stakeholders directly or indirectly involved in wildlife and wildlife products is imperative. Mainly, the operationalization of the framework is fund-dependent, and it requires community cooperation. Hence, it is important to highlight Community Resource Boards (CRBs), which are identified in the Zambia Wildlife Act of 2015, as institutions for communities to co-manage and benefit from the GMA. In terms of SI 89 of 2004, 50% of animal license fees are allocated to (CRBs) for community development, out of which 5% go directly to the chief. Regarding CRB, one informant reported that:

*“Additionally, they have the community resource boards, yes, they have them, but they are there on paper, nothing is being implemented, still the management is coming from top to down, that is what I found during my PhD research, that was my area of focus. During the time I was collecting my data, people were saying it has been 4 years now since the establishment of the CRBs, and we have not received anything from the 50% we should get from the trophy hunting from the government. So that is the challenge, we do not apply what is in the policy documents, no funds reach the community despite the act stipulating that the community should receive a certain percentage. The challenge, I think, is in most African countries; it may not be only in Zambia. My colleague at some point said they have the same challenge in Kenya”.*

A case study by Snyman and Mwale [126] reports that the total annual disbursements from animal fees and hunting concession agreement concession fees in 2016 and 2017 were approximately USD 1 million/year. Each CRB, on average, received approximately USD 14,000/year, although this distribution was unequal. The report further states that the CRBs are encouraged to spend these funds in the following ratios: law enforcement, 45%; community projects, 35%; and administrations, 20%; however, in general, the CRBs can decide how the funds are used. There is a need to further investigate whether the funds are reaching the communities or all the communities. Whether funds are reaching the communities or not, the role of CRBs is an important factor in the operation of the framework as it links the community and the wildlife resources.

The study makes these specific recommendations for the operationalization of the framework. The community should be involved in decision-making; they should be part of the conversation, and before any intervention, it is important to perform a needs assessment. The government should commit to the 50% animal license fees revenues payable to the community fund. The fund can promote awareness campaigns on the importance of education so that children can see the importance of education, school feeding programs to encourage children to go to school, and scholarships for children who may end up not being enrolled in state-funded institutions. Establish community industries using the fund to provide work for poachers and motivate children to go to school, given that there are employment opportunities after school. They should be a balance in the use of funds between enforcement efforts and education programs. The state should systematically cull wildlife and provide game meat to locals for subsistence use rather than just waiting for trophy hunters or reducing license fees for locals during hunting seasons. The government and partners should increase resources (financial and human) for the DNPW; run community awareness campaigns on the ecological, legal, and health risks of illegal wild meat; provide wildlife conservation education in rural schools; and provide training and start-up support for game farming. Several ministries and permits are involved in game farming and game meat selling in Zambia; it is important to create one department that deals with the regulations and permits or just simplify and incentivize the licensing process for captive breeding.

## 5. Limitations

The study could have benefited from poacher interviews. However, owing to the illegality of the wild meat trade in Zambia, people do not open up about poaching and poachers and illegal wild meat trade intermediaries, as people are afraid of being arrested; even if you explain that you are researchers, they think you are tricking them. Other limitations include the lack of quantitative data to measure offtake and species type and the fact that interviews were mainly conducted with conservationists and law enforcement personnel (no consumers or poachers). The risk questions were not specific to pathogens or species.

## 6. Conclusions

Unhygienic practices and poor handling practices were identified along the supply chain. Misrepresentation of species and selling of dead or sick animals are also identified. There is high demand for illegal game meat. The focus should be persuading consumers to stop purchasing illegally traded meat and other actors in the supply chain to first avoid illicit trade and shift toward safe, sustainable, and legal behaviors while calling on governments and private sector actors to increase their management and monitoring. There is a need to empower local traditional institutions such as chiefs, headmen, and village heads and their structures to regulate wild meat extraction. Government and non-governmental organizations should develop policies that promote alternative, sustainable livelihoods for communities living near game management areas. Given the similarities in the illegal wildlife trade, the findings of this study can help with a better understanding of the risks associated with unlawful wild meat and shape future interventions to reduce wild meat risks.
